# Supplementary material for: Telomerase RNA component knockout exacerbates Staphylococcus aureus pneumonia by extensive inflammation and dysfunction of T cells
Source: eLife. 2024 Nov 28;13:RP100433. doi: 10.7554/eLife.100433 (PMC11604217; doi:10.7554/eLife.100433)

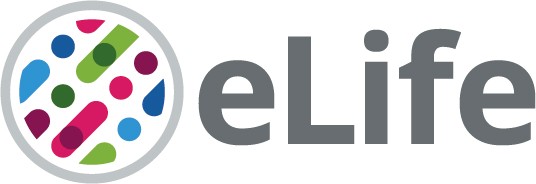

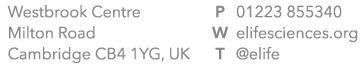


Materials Design Analysis Reporting (MDAR)

Checklist for Authors

The MDAR framework establishes a minimum set of requirements in transparent reporting mainly applicable to studies in the life sciences.

*eLife* asks authors to **provide detailed information within their article** to facilitate the interpretation and replication of their work. Authors can also upload supporting materials to comply with relevant reporting guidelines for health- related research (see EQUATOR Network), life science research (see the BioSharing Information Resource), or animal research (see the ARRIVE Guidelines and the STRANGE Framework; for details, see *eLife*’s Journal Policies). Where applicable, authors should refer to any relevant reporting standards materials in this form.

For all that apply, please note **where in the article** the information is provided. Please note that we also collect information about data availability and ethics in the submission form.

**Materials:**

| **Newly created materials** | **Indicate where provided: section/figure legend** | **N/A** |
| --- | --- | --- |
| The manuscript includes a dedicated "materials availability statement" providing transparent disclosure about availability of newly created materials including details on how materials can be accessed and describing any restrictions on access. |  | N/A |

| **Antibodies** | **Indicate where provided: section/figure legend** | **N/A** |
| --- | --- | --- |
| For commercial reagents, provide supplier name, catalogue number and RRID, if available. | Information can be found in Material and Methods Section, under “Histology and Immunofluorescence”, “Immunocytochemistry”, “Immunophenotyping of the spleen” and “Flow cytometry” |  |

| **DNA and RNA sequences** | **Indicate where provided: section/figure legend** | **N/A** |
| --- | --- | --- |
| Short novel DNA or RNA including primers, probes: Sequences should be included or deposited in a public repository. | Information can be found in Material and Methods Section, under “Mouse models and infection” |  |

| **Cell materials** | **Indicate where provided: section/figure legend** | **N/A** |
| --- | --- | --- |
| Cell lines: Provide species information, strain. Provide accession number in repository OR supplier name, catalog number, clone number, OR RRID. |  | N/A |
| Primary cultures: Provide species, strain, sex of origin, genetic modification status. | Information can be found in Material and Methods Section, under “Isolation and infection of murine T cells and alveolar macrophages” |  |

| **Experimental animals** | **Indicate where provided: section/figure legend** | **N/A** |
| --- | --- | --- |
| Laboratory animals or Model organisms: Provide species, strain, sex, age, genetic modification status. Provide accession number in repository OR supplier name, catalog number, clone number, OR RRID. | Information can be found in Material and Methods Section, under “Mouse models and infection” |  |
| Animal observed in or captured from the field: Provide species, sex, and age where possible. |  | N/A |

| **Plants and microbes** | **Indicate where provided: section/figure legend** | **N/A** |
| --- | --- | --- |
| Plants: provide species and strain, ecotype and cultivar where relevant, unique accession number if available, and source (including location for collected wild specimens). |  | N/A |
| Microbes: provide species and strain, unique accession number if available, and source. | Information can be found in Material and Methods Section, under “Bacteria” |  |

| **Human research participants** | **Indicate where provided: section/figure legend) or state if**  **these demographics were not collected** | **N/A** |
| --- | --- | --- |
| If collected and within the bounds of privacy constraints report on age, sex, gender and ethnicity for all study participants. |  | **N/A** |

**Design:**

| **Study protocol** | **Indicate where provided: section/figure legend** | **N/A** |
| --- | --- | --- |
| If the study protocol has been pre-registered, provide DOI. For clinical trials, provide the trial registration number OR cite DOI. |  | N/A |

| **Laboratory protocol** | **Indicate where provided: section/figure legend** | **N/A** |
| --- | --- | --- |
| Provide DOI OR other citation details if detailed step-by-step protocols are available. |  | N/A |

| **Experimental study design (statistics details)** | | |
| --- | --- | --- |
| **For in vivo studies: State whether and how the following have been done** | **Indicate where provided: section/figure legend. If it could have been done, but was not, write “not done”** | **N/A** |
| Sample size determination | Based on statistical assesement of the animal testing application | N/A |
| Randomisation | Not done |  |
| Blinding | Not done |  |
| Inclusion/exclusion criteria | Information can be found in Material and Methods Section, under “Mouse models and infection” |  |

| **Sample definition and in-laboratory replication** | **Indicate where provided: section/figure legend** | **N/A** |
| --- | --- | --- |
| State number of times the experiment was replicated in the laboratory. | Information can be found within Figure Legends |  |
| Define whether data describe technical or biological replicates. | Information can be found within Figure Legends |  |

| **Ethics** | **Indicate where provided: section/submission form** | **N/A** |
| --- | --- | --- |
| Studies involving human participants: State details of authority granting ethics approval (IRB or equivalent committee(s), provide reference number for approval. |  | N/A |
| Studies involving experimental animals: State details of authority granting ethics approval (IRB or equivalent committee(s), provide reference number for approval. | Information can be found within Materials and Methods in “Animal Studies” |  |
| Studies involving specimen and field samples: State if relevant permits obtained, provide details of authority approving study; if none were required, explain why. |  | N/A |

| **Dual Use Research of Concern (DURC)** | **Indicate where provided: section/submission form** | **N/A** |
| --- | --- | --- |
| If study is subject to dual use research of concern regulations, state the authority granting approval and reference number for the regulatory approval. |  | N/A |

**Analysis:**

| **Attrition** | **Indicate where provided: section/figure legend** | **N/A** |
| --- | --- | --- |
| Describe whether exclusion criteria were pre-established. Report if sample or data points were omitted from analysis. If yes, report if this was due to attrition or intentional exclusion and provide justification. | Information can be found within Figure Legends |  |

| **Statistics** | **Indicate where provided: section/figure legend** | **N/A** |
| --- | --- | --- |
| Describe statistical tests used and justify choice of tests. | Information can be found within Figure Legends and within Materials and Methods “Statistical analysis and scheme design” |  |

| **Data availability** | **Indicate where provided: section/submission form** | **N/A** |
| --- | --- | --- |
| For newly created and reused datasets, the manuscript includes a data availability statement that provides details for access (or notes restrictions on access). | Information can be found within Figure Legends Materials and Methods in “RNA-seq Analysis” and “RNA extraction and mRNA sequencing” as well as in the Data availability statement |  |
| When newly created datasets are publicly available, provide accession number in repository OR DOI and licensing details where available. | Information can be found within Data availability statement |  |
| If reused data is publicly available provide accession number in repository OR DOI, OR URL, OR citation. |  | N/A |

| **Code availability** | **Indicate where provided: section/figure legend** | **N/A** |
| --- | --- | --- |
| For any computer code/software/mathematical algorithms essential for replicating the main findings of the study, whether newly generated or re-used, the manuscript includes a data availability statement that provides details for access or notes restrictions. |  | N/A |
| Where newly generated code is publicly available, provide accession number in repository, OR DOI OR URL and licensing details where available. State any restrictions on code availability or accessibility. |  | N/A |

| If reused code is publicly available provide accession number in repository OR DOI OR URL, OR citation. |  | N/A |
| --- | --- | --- |

**Reporting:**

The MDAR framework recommends adoption of discipline-specific guidelines, established and endorsed through community initiatives.

| **Adherence to community standards** | **Indicate where provided: section/figure legend** | **N/A** |
| --- | --- | --- |
| State if relevant guidelines (e.g., ICMJE, MIBBI, ARRIVE, STRANGE) have been followed, and whether a checklist (e.g., CONSORT, PRISMA, ARRIVE) is provided with the manuscript. | Animal experiments were conducted in accordance with the German and Thuringian Animal Welfare Regulations. | N/A |


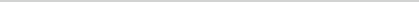

Supplement: MDAR checklist [file elife-100433-mdarchecklist1.docx]
