# Supplementary figures and images for: Telomerase RNA component knockout exacerbates Staphylococcus aureus pneumonia by extensive inflammation and dysfunction of T cells
Source: eLife. 2024 Nov 28;13:RP100433. doi: 10.7554/eLife.100433 (PMC11604217; doi:10.7554/eLife.100433)

## Slide 1
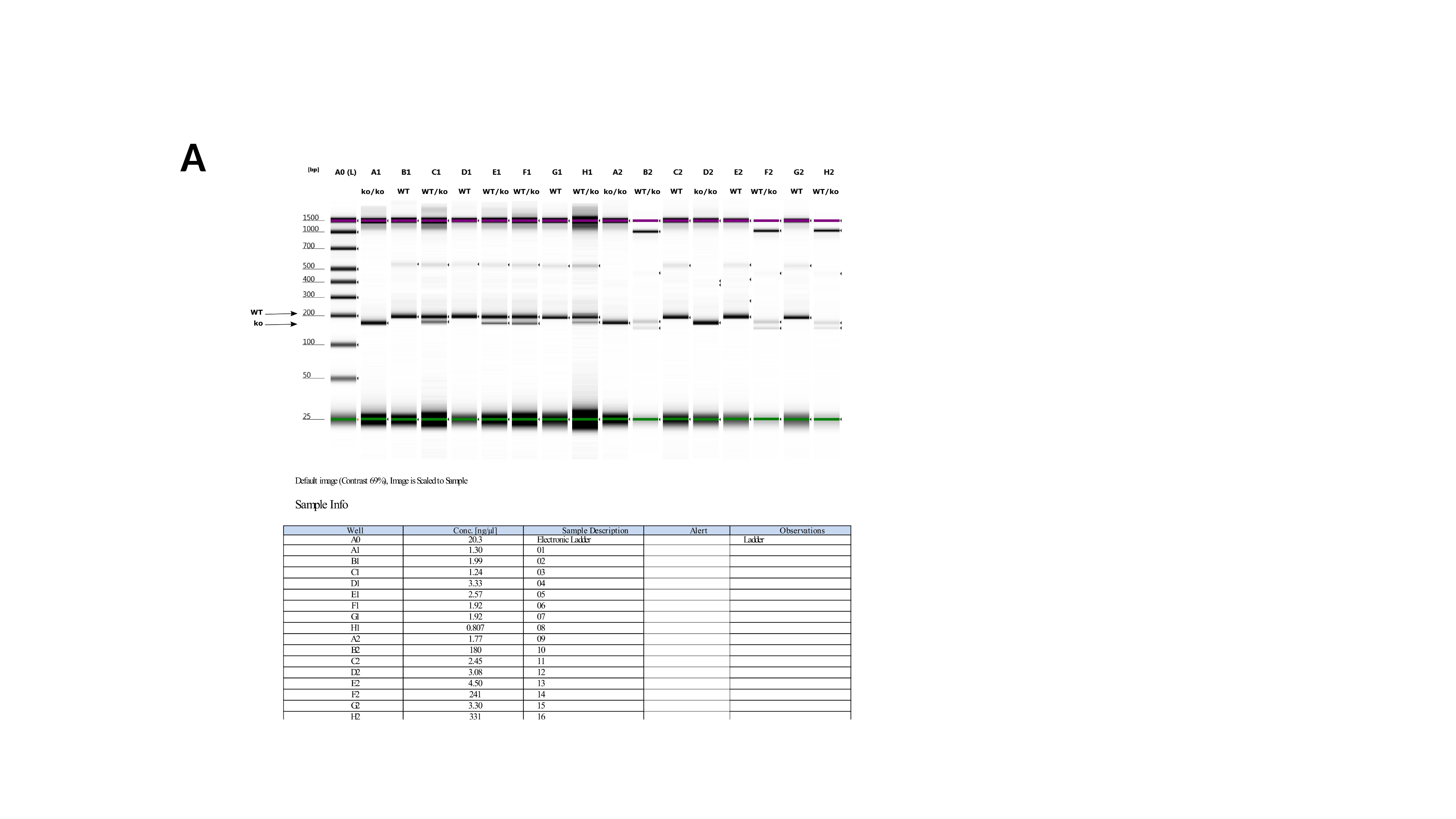

A

Supplement: Supplementary file 4. [file elife-100433-supp4.pptx]

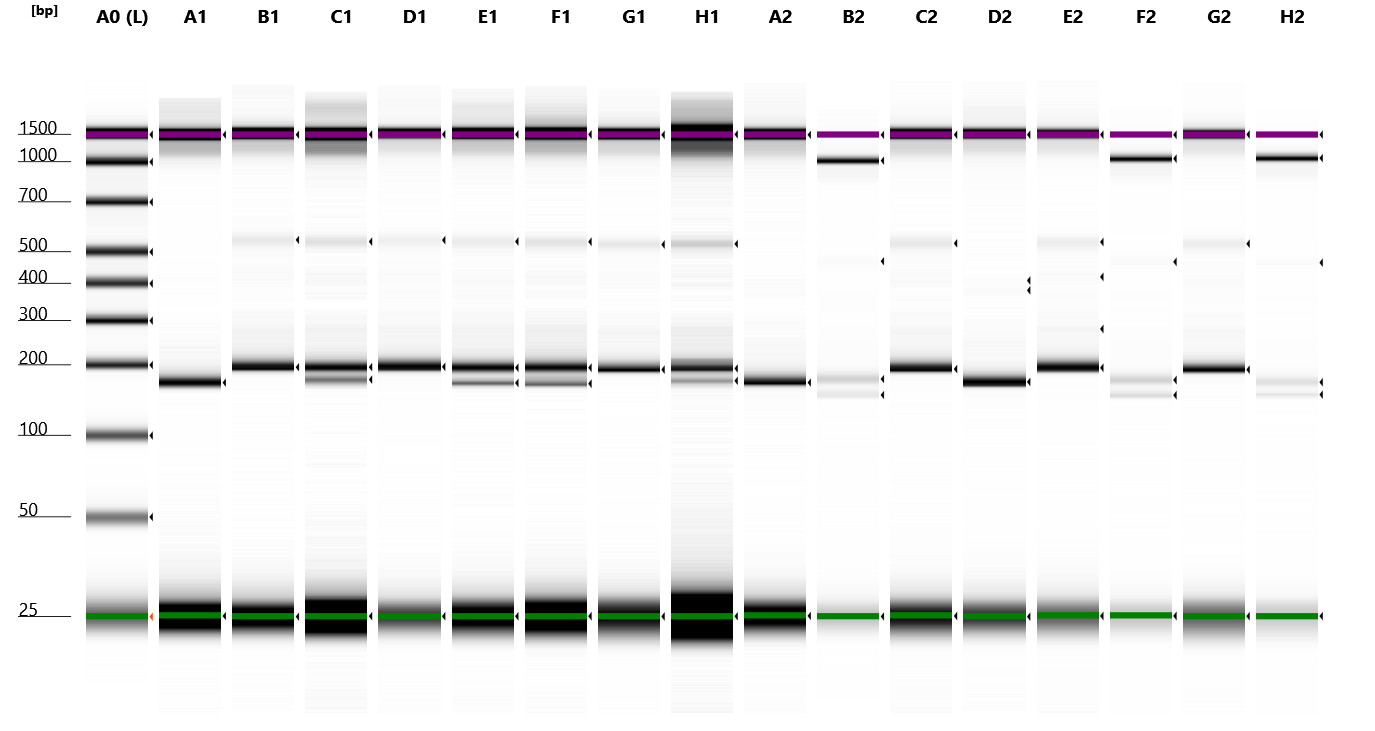

Supplement: Source data 1. [file elife-100433-data1.zip › Supplementary File 4-Source Data 1.png]

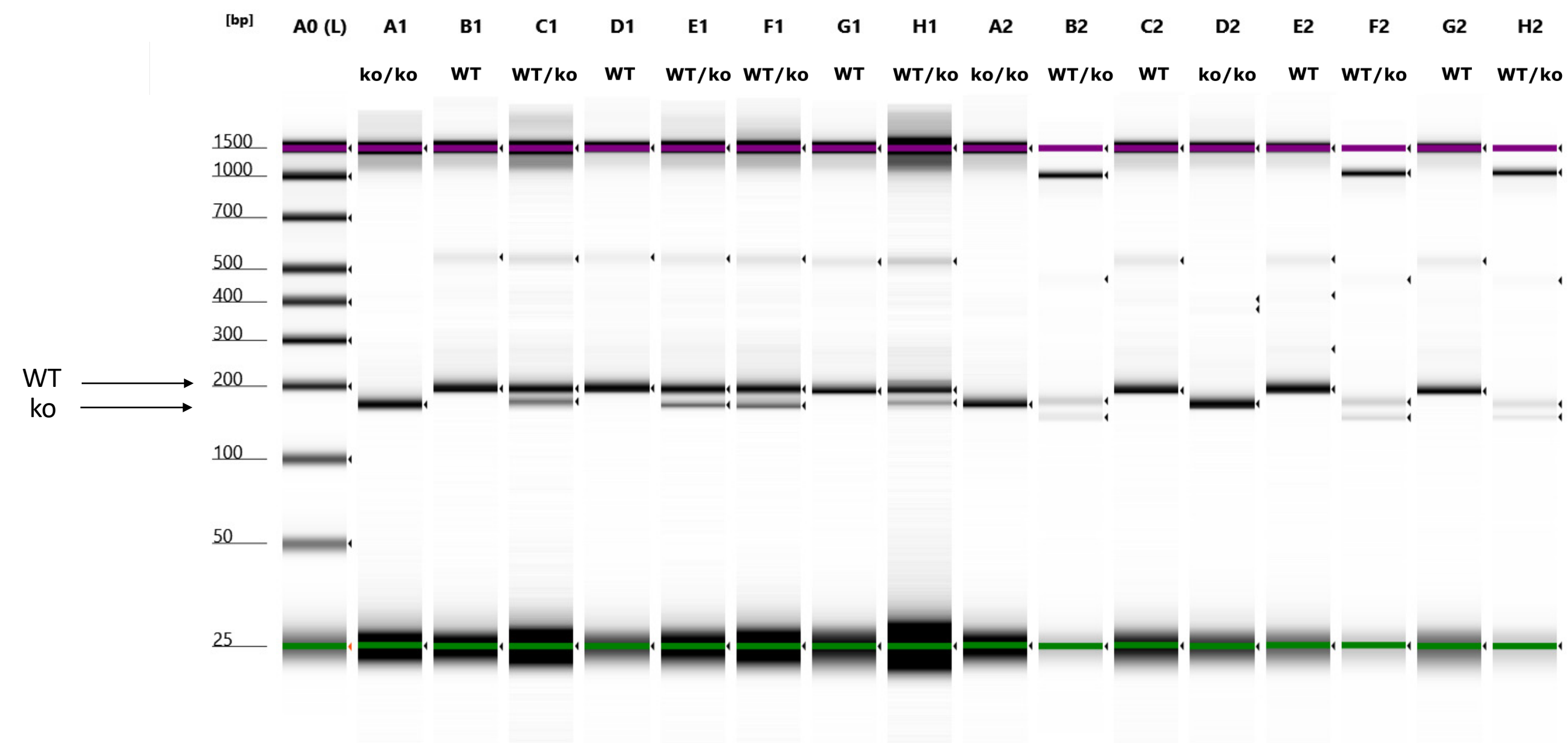

Supplement: Source data 2. [file elife-100433-data2.zip › Supplementary File 4 - Source data 2/Supplementary File 4-Source Data 2.pdf]
